# Elevated fasting glucose and triglycerides in combined oral contraceptive users compared to naturally cycling females

**DOI:** 10.14814/phy2.71135

**Published:** 2026-10-12

**Authors:** Claire Karaguesian, Jennifer S. Williams, Maureen J. MacDonald, Kirsten E. Bell

**Affiliations:** ^1^ Department of Kinesiology McMaster University Hamilton Ontario Canada

**Keywords:** body composition, females, hormones, insulin resistance, lean mass

## Abstract

The effects of oral contraceptive pills (OCPs) on metabolism and body composition remain unclear. This study aimed to compare fasting glucose and lipid metabolism between naturally cycling (NAT) females, second‐(OCP2), and third‐generation (OCP3) OCP users during low‐(LH) and high‐hormone (HH) phases. Forty‐nine healthy females (age: 21 ± 2 years, BMI: 23.5 ± 3.8 kg/m^2^) were assessed. Participants were assessed in two randomized fasting blood draws during: (1) LH phase (NAT: early follicular, OCP: placebo pill week), and (2) HH phase (NAT: mid‐luteal, OCP: third week of active pill use) to assess glucose, insulin, and lipids. Dual‐energy X‐ray absorptiometry was performed to assess body composition. Glucose was higher in OCP2 (4.4 ± 0.3 mM, *p* = 0.002) and OCP3 (4.5 ± 0.3 mM, *p* < 0.001), compared to NAT (4.0 ± 0.5 mM), with no effect of hormone phase (*p* = 0.895); insulin was not different across groups or phases. Triglycerides were higher in OCP2 (0.9 ± 0.5 mM, *p* = 0.036) and OCP3 (0.9 ± 0.3 mM, *p* = 0.011), compared to NAT (0.6 ± 0.2 mM). Appendicular lean mass index was lower in OCP2 (6.5 ± 0.8 kg/m^2^, *p* = 0.030) and OCP3 (6.5 ± 0.5 kg/m^2^, *p* = 0.030) compared to NAT (7.2 ± 1.1 kg/m^2^). Findings suggest that OCP use associates with a distinct metabolic profile, potentially related to differences in lean mass, an important glucose sink.

## 1 INTRODUCTION

Approximately 1 in 5 Canadians meet the criteria for metabolic syndrome (MetS) (Dev et al., 2024), a cluster of interconnected symptoms (including high waist circumference, blood pressure, and fasting glucose; and unfavorable lipid partitioning) that increase risk of cardiovascular disease and type 2 diabetes (T2D) (Alberti et al., 2009; Cornier et al., 2008). However, given that MetS is often underdiagnosed (Fujiyoshi et al., 2011), its true prevalence may be higher. Females may be particularly underrepresented in prevalence statistics since public messaging around the dangers of cardiovascular disease and T2D, and the need for biomarker screening, has traditionally targeted males (Damaskos et al., 2020). The historic lag in research on female physiology has also likely contributed to the emphasis on male risk for cardiometabolic disorders. Over the past 20 years, deliberate efforts have been made to increase female representation in research (Reusch et al., 2018), but our understanding of cardiometabolic health indicators for females remains incomplete (Clavel et al., 2024).

In Canada, 75% of women will use combined oral contraceptive pills (OCPs) at some point in their lives, with second‐ (OCP2) and third‐generation (OCP3) OCPs being most commonly prescribed (Black et al., 2015; Rotermann et al., 2015). Yet, despite their widespread use, the effect of OCPs on indicators of metabolic health is unclear, with some studies reporting a beneficial association between OCP use and features of glucose and lipid metabolic health (Kim et al., 2002; Troisi et al., 2000), others demonstrating a negative association (Frempong et al., 2008; Morin‐Papunen et al., 2008), and others failing to detect a relationship (Isacco et al., 2014; Nessa et al., 2005; Straznicky et al., 1998). One possible explanation for these discrepant findings is that some studies were prospective interventions that compared participants before and 2–3 months after starting an OCP (Beasley et al., 2012; Piltonen et al., 2012; Sheu et al., 1994), while others were cross‐sectional observation studies that compared longer‐term OCP users (2–3+ years) to naturally cycling individuals (Frempong et al., 2008; Isacco et al., 2014; Kim et al., 2002; Morin‐Papunen et al., 2008; Nessa et al., 2005; Straznicky et al., 1998; Troisi et al., 2000). Given that physicians often recommend patients wait 3–5 pill cycles after starting a new form of hormonal contraception for potential adverse effects (e.g., headaches, mood alterations, and breakthrough bleeding) to subside (Grossman Barr, 2010), it is reasonable to assume that the body habituates to OCP use over time. Therefore, it may not be appropriate to compare certain physiological outcomes in shorter versus longer‐term OCP users. Another potential explanation is that not all previous studies included a control group of naturally cycling individuals (Beasley et al., 2012; Piltonen et al., 2012; Sheu et al., 1994), making it difficult to attribute observed differences in metabolism to either the OCPs themselves or to natural variation. Lastly, even though hormone concentrations vary considerably throughout the OCP (Rodriguez et al., 2024) and menstrual cycles (Yeung et al., 2010), many studies failed to report (Beasley et al., 2012; Nessa et al., 2005; Piltonen et al., 2012; Straznicky et al., 1998), or properly control for (Frempong et al., 2008), the pill or cycle day on which measurements were obtained or the generation of OCP used. Thus, while estrogen and progesterone (and their synthetic versions) may (Escalante Pulido & Alpizar Salazar, 1999; González‐Ortiz et al., 1998; Yeung et al., 2010) or may not (Bingley et al., 2008; Blum et al., 2005; Cree et al., 2024) influence insulin sensitivity and lipid partitioning, more carefully controlled work is needed.

OCP use is not associated with significant weight gain (Berenson et al., 2008; Berenson & Rahman, 2009; Gallo et al., 2004; Moreau et al., 2007), but the effects of OCPs on body composition (i.e., the quantity and anatomical distribution of fat and lean tissues) are unclear. To our knowledge, no study has tested for differences in body composition between OCP users and naturally cycling females. Recently, Kuikman et al. (2024) observed that young female athletes using hormonal contraception (including, but not limited to, OCPs) had higher whole‐body lean mass by dual‐energy X‐ray absorptiometry (DXA) compared with naturally cycling athletes. In contrast, Suuronen et al. (2019) observed in a large sample of generally healthy Finnish women aged 20–40 years that hormonal contraceptive users (including OCP, progesterone‐only pill, implant, and intrauterine device users) had lower whole‐body lean mass by DXA compared to naturally cycling individuals. Importantly, neither of these studies tested for between‐group differences in key regional depots known to impact metabolic health. For example, leg and gynoid fat mass are protective against hypertriglyceridemia in younger and middle‐aged adults, whereas upper body fat mass is associated with an increased risk for type‐2 diabetes and cardiovascular disease (Vasan et al., 2018). Given that the quantity and anatomical distribution of adipose tissue (Goodpaster et al., 2005) and skeletal muscle (Bell et al., 2022) are significant determinants of glucose‐associated metabolic health, we need to improve our understanding of the potential interaction between OCP use and body composition.

The objectives of this study were to determine the effects of the natural menstrual cycle (NAT) and commonly prescribed OCPs (OCP2 and OCP3) on features of glucose (primary objective) and lipid (secondary objective) metabolism, and body composition (tertiary objective) in young, healthy females. We hypothesized that features of glucose metabolism and lipid partitioning would be less favorable in OCP2 and OCP3 users compared with NAT females, as evidenced by higher fasting concentrations of glucose, insulin, triglycerides, total cholesterol, and low‐density lipoprotein cholesterol, and lower concentrations of high‐density lipoprotein cholesterol in the OCP user NAT females. We further hypothesized that these features of glucose and lipid metabolism would be unaffected by the low hormone (LH, early follicular or during placebo pill use) and high hormone (HH, mid‐luteal or during active pill use) phases. Lastly, we hypothesized that neither OCP use nor hormone phase would affect body composition as indicated by whole‐body or regional fat and lean soft tissue mass.

## 2 MATERIALS AND METHODS

### 2.1 Participants

This study was part of a larger research project that examined the effect of OCP and sex hormone fluctuations on vascular outcomes (Williams et al., 2024). Forty‐nine healthy females aged 18–45 years were recruited from McMaster University and the greater Hamilton, Ontario community between 2020 and 2022 and subsequently allocated into groups based on OCP usage: (1) natural menstrual cycle (NAT group, *n* = 17); second generation combined oral contraceptive pill users (OCP2 group, *n* = 17); and third generation combined oral contraceptive pill users (OCP3 group, *n* = 15). Individuals in the NAT group were excluded if their menstrual cycles were irregular (i.e., 1 missed cycle in the previous 6 months) or had durations outside of the defined “normal” range (24–38 days per cycle) or if they had taken any form of hormonal contraception in the previous 6 months. Participants in the NAT group tracked their menstrual cycles for at least 2 months before visit 1 to allow researchers to calculate the average cycle length, defined as duration between the onset of menses between two subsequent cycles. Alongside menstrual cycle length, we also objectively assessed the timing of ovulation and sex hormone concentrations in accordance with current guidelines on menstrual cycle verification (which we describe in more detail in the next paragraph) (Elliott‐Sale et al., 2025). Individuals in the OCP2 and OCP3 groups were taking either monophasic or triphasic formulations for a minimum of 3 months before beginning the study and were excluded if they did not use the medication as prescribed (e.g., skipping the placebo pill phase). Regardless of group, individuals were excluded from the study if they were pregnant or breastfeeding within the last year, had cardiovascular or metabolic disease, regularly used vasoactive medications or tobacco products, or had undergone reproductive surgeries (e.g., oophorectomy and hysterectomy) or had reproductive conditions. For additional details concerning participant eligibility and the menstrual cycle/pill use, we refer readers to the original (Williams et al., 2024) and secondary (Williams et al., 2023) publications. This study conforms to the standards set by the Declaration of Helsinki, apart from registration as a clinical trial, and was approved by the Hamilton Integrated Research Ethics Board (HIREB #7827). Written informed consent was obtained from all volunteers prior to their participation. Separate consent to publish an image was obtained from the individual depicted Figure 2.

### 2.2 Experimental design

After a baseline assessment (for anthropometrics and VO_2_peak), participants reported to the laboratory for 2 study visits that were scheduled to coincide with their low (LH) and high (HH) phases (Figure 1). The order of these visits was assigned and counterbalanced within each group (NAT, OCP2, and OCP3) such that approximately half the participants per group attended visit 1 in their LH phase and the other half attended visit 1 in their HH phase. For the NAT group, we defined the LH phase as the early follicular phase of the menstrual cycle (days 3–7, where the onset of menses is day 1), and the HH phase as the mid‐luteal phase (~7 days post‐ovulation). Ovulation was confirmed by participants using standard at‐home ovulation kits (BFP Ovulation Tests, Fairhaven Health; Bellingham, WA) prior to the HH phase visit. For the OCP2 and OCP3 groups, we defined the LH phase as days 3–7 of placebo pill use and the HH phase as days 2–7 of the third week of active pill use. This ensured that participants on triphasic pills attended their HH phase visit during their highest active dose week. During each visit, a single fasting blood sample was collected. During the LH phase visit, a whole‐body dual‐energy X‐ray absorptiometry (DXA) scan was also conducted.

**FIGURE 1:**
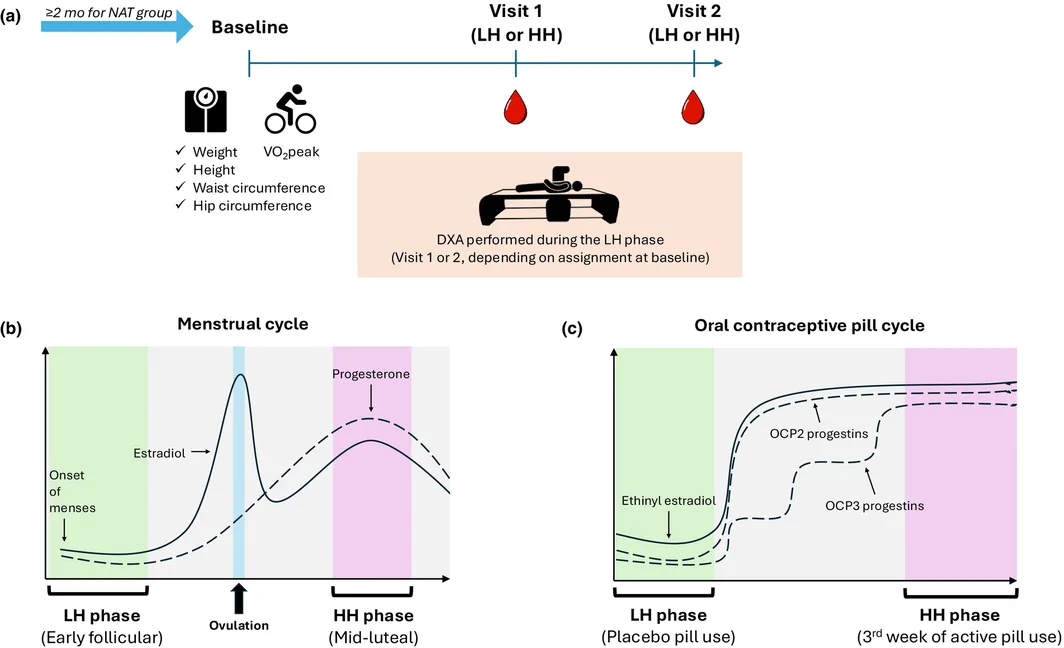
Overview of experimental design (a) and typical hormone fluctuations associated with the menstrual cycle (b) and oral contraceptive pill cycle (c). The solid lines represent estradiol (in the NAT group) or ethinyl estradiol (OCP users). The dotted lines represent progesterone (NAT group) or progestins (OCP users). After a baseline assessment, all participants attended two study visits that coincided with either their LH or HH phases. A single fasted blood draw was performed during each visit, and a whole‐body DXA scan was performed during the LH visit. DXA, dual‐energy X‐ray absorptiometry; HH, high hormone; LH, low hormone; NAT, naturally cycling; OCP2, second‐generation oral contraceptives; OCP3, third‐generation oral contraceptives. Figure created in Microsoft Powerpoint.

### 2.3 Baseline assessment

Weight and height were measured using a scale and stadiometer, respectively. Body mass index (BMI) was calculated as weight (kg)/height (m)^2^, with participants classified into BMI categories as follows: <18.5 kg/m^2^ (underweight), 18.5–24.9 kg/m^2^ (normal weight), 25.0–29.9 kg/m^2^ (overweight), 30.0–34.9 kg/m^2^ (obesity class I), ≥35.0 kg/m^2^ (obesity class II) (Canada H, 2021). Waist and hip circumferences were measured at the top of the iliac crests and the widest portion of the hips, respectively, using a flexible tape measure while participants stood with their feet hip‐width apart. Waist‐to‐hip ratio was calculated as waist circumference/hip circumference. Blood pressure was assessed in the supine position after 10 min of rest. We report the average of the last two of three measurements obtained with an automated BP assessment device (GE Dinamap ProSeries, Batesville, IN). If the second and third systolic blood pressure measurements were not within 5 mmHg of each other, a fourth measurement was obtained, and the last two measures were averaged. Systolic (SBP) and diastolic (DBP) were reported.

Participants then performed a VO_2_peak test on an upright cycle ergometer (Lode Excalibur Sport V 2.0; Groningen, Netherlands or Kettler Ergo Race; Virginia Beach, VA) while wearing a chest‐strap heart rate monitor (Polar A3; Lake Success, NY). A metabolic cart and online gas collection system (Quark CPET Metabolic Cart; COSMED, Italy) were used to quantify respiratory gases. Following a 3‐min warm‐up at 50 W, the load was increased by 1 W every 2 s (*n* = 37) or 5 W every 10 s (*n* = 12), depending on the cycle ergometer used. Participants were instructed to maintain a cadence of at least 60 rpm. Tests were terminated if: (1) cadence dropped below 60 rpm for >10 s, (2) heart rate was within 10 bpm of 208–0.7*age, (3) RER was >1.1, (4) we observed a clear plateau in VO_2_, or (5) a participant's perceived exertion was ≥17 (Borg scale 6–20). All participants achieved VO_2_peak, based on these criteria.

### 2.4 Blood sampling

Participants were instructed to refrain from vigorous physical activity for >24 h and food, alcohol, caffeine, and nonprescription medications (e.g., anti‐inflammatory and pain medication) for 8–12 h prior to testing. Blood draws were always scheduled during the morning to midday hours and at the same time of day for a given participant. After a 10 min rest in the supine position, approximately 30 mL of venous blood was collected into evacuated blood collection tubes by a trained phlebotomist. The blood sample was allowed to clot at room temperature for ~60 min prior to centrifugation at 4000 rpm at 4°C for 10 min. The serum was then aliquoted into 1.5 mL polypropylene tubes and frozen for batch analysis.

### 2.5 Dual‐energy X‐ray absorptiometry

Whole body and regional lean soft tissue mass (i.e., fat‐ and bone‐free mass), fat mass, and bone mineral density were measured using DXA (GE‐LUNAR iDXA; GE, Mississauga, ON). Quality control and calibration procedures using phantoms provided by the manufacturer were performed daily prior to performing the scan. After participants changed into light clothing and removed all metal (e.g., jewelry and piercings), weight was obtained using a scale. Participants were then positioned supine on the scanning table with their spine aligned with the middle of the bed, shoulders depressed, forearms positioned parallel to the bed, and feet together for the scan. LUNAR iDXA software (v18.0) automatically segments whole‐body scans into the head, trunk, left and right arms, and left and right legs. A single study investigator (CK) verified the segmentation according to a standardized protocol and adjusted regions of interest as needed. The analyst was blinded to the group, and scans from each group were analyzed in a counterbalanced manner. Appendicular lean mass (in kg/m^2^) was calculated by summing the lean soft tissue mass of all four limbs and dividing by height squared. Android and gynoid fat mass was analyzed using the automated analysis included in the iDXA software package. The lower boundary of the android region began at the top of the iliac crests (horizontal pelvic line), and the upper boundary was located 20% of the distance between the horizontal pelvic line and the neck cut line (Figure 2). The height of the gynoid region was twice the height of the android region. The upper boundary of the gynoid region began below the horizontal pelvic line at a distance equal to 1.5 times the height of the android region. The leg cut lines were the lateral limits of the gynoid region. The android/gynoid (A/G) ratio was calculated by dividing the android fat mass by the gynoid fat mass.

**FIGURE 2:**
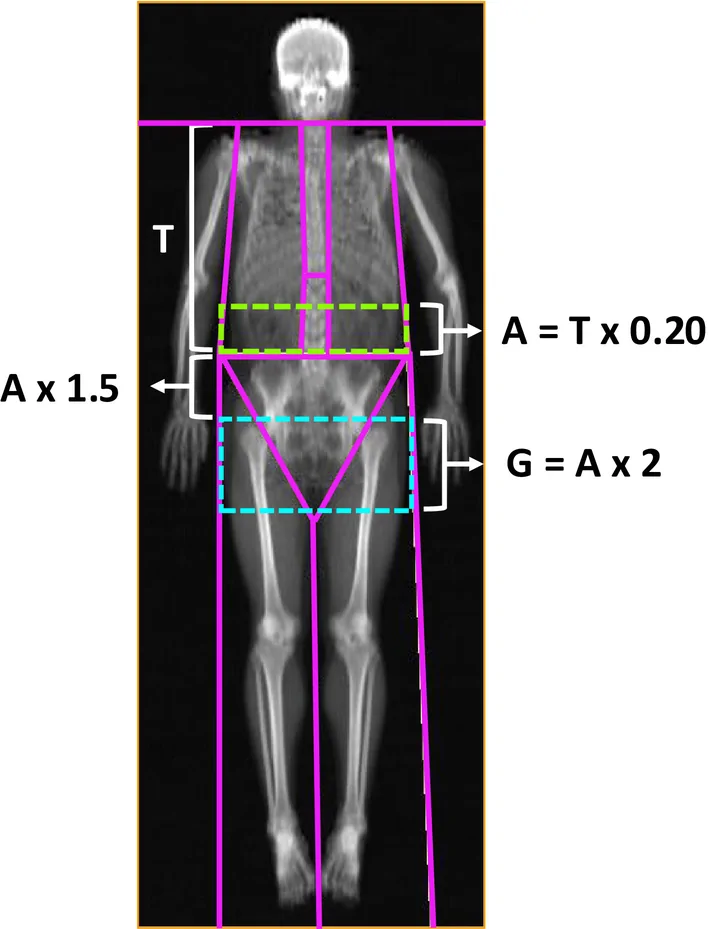
Landmarks for DXA regional analysis. The purple lines indicate the boundaries between the head, trunk (which included the spine and pelvis), left and right arms, and left and right legs. The android region is indicated by the dashed green box, and the gynoid region is indicated by the dashed blue box. The white letter “T” represents the distance between the horizontal pelvic line and the neck cut line. The height of the android region (“A”) was calculated as 20% of the length of “T”. The horizontal pelvic line was the lower boundary of the android region. The height of the gynoid region was twice the height of the android region. The upper boundary of the gynoid region began below the horizontal pelvic line at a distance equal to 1.5 times “A”. The lateral limits of the android and gynoid regions were the arm and leg cut lines. This image is a representative image from our data set. Consent to publish an image was obtained from the individual depicted.

### 2.6 Biochemical analysis

Glucose was measured spectrophotometrically using an in‐house glucose oxidase assay. Insulin was measured using an enzyme‐linked immunosorbent assay kit (Alpco Diagnostics Cat# 80‐INSHU‐E01.1, RRID:AB_2801438). The homeostatic model assessment of insulin resistance (HOMA‐IR) was calculated using the following equation:

$$\text{HOMA}‐\mathrm{IR}=\frac{\text{Fasting glucose}\;\left(\text{in}\;\frac{\text{mmol}}{L}\right)x\;\text{Fasting insulin}\;\left(\text{in}\;\frac{\mathrm{\mu IU}}{\mathrm{mL}}\right)}{22.5}$$

Total cholesterol (TC) and triglycerides (TAG) were measured with commercially available kits (BioPacific Diagnostic Inc.; Vancouver, BC), and high‐density lipoprotein cholesterol (HDL‐c) was measured by the Hamilton Regional Laboratory Medicine Program at McMaster University Medical Centre using a direct HDL‐c slide analysis (VITROS Chemistry Products). Low‐density lipoprotein cholesterol (LDL‐c) was calculated using the Friedewald equation (Friedewald et al., 1972):

$$\mathrm{LDL}‐c=\mathrm{TC}-\mathrm{HDL}‐c-\frac{\mathrm{TAG}}{2.2}$$

All samples were analyzed in duplicate. Coefficients of variation for assays performed in our lab were <5% (for glucose, TC, and TAG) or <10% (for insulin).

### 2.7 Statistical analysis

Statistical analyses were performed in SPSS v27.0, with significance set at α < 0.05. Data are presented as mean ± standard deviation (SD) unless otherwise indicated.

One‐way ANOVAs were used to test for between‐group differences in age, anthropometrics, regional and whole‐body DXA outcomes, and VO_2_peak outcomes. Blood metabolites (glucose, insulin, and lipid panel) were analyzed using 2‐way mixed‐design ANOVAs with group (NAT vs. OCP2 vs. OCP3) as the between‐subjects factor and hormone phase (LH vs. HH) as the within‐subject factor. In the case of significant F‐ratios, specific between‐group differences were identified using Tukey's posthoc test. For differences that trended towards statistical significance (0.05 < *p* < 0.10), we calculated and reported the associated effect sizes (eta‐squared or *η* ^2^). According to recommendations by Cohen (1988), effect sizes of 0.01, 0.06, and 0.14 were considered small, medium, and large, respectively. A *Χ* ^2^ test was used to examine whether the proportion of participants across BMI categories differed between groups.

In an exploratory sub‐analysis, we used Pearson correlations to test for associations between appendicular lean mass index (ALMI) and fasting glucose concentrations in the LH and HH phases when all participants were pooled into a single group. Based on recommendations by Schober et al. (2018), significant correlation coefficients (*r*) were considered negligible (0.00–0.10), weak (0.10–0.39), moderate (0.40–0.69), strong (0.70–0.89), or very strong (0.90–1.00).

## 3 RESULTS

### 3.1 Participant characteristics

Forty‐nine healthy young females participated in the study (NAT: *n* = 17, OCP2: *n* = 17, OCP3: *n* = 15), and details concerning their physical characteristics, body composition, and menstrual cycle/contraceptive pill use can be found in Table 1. All participants in the OCP2 group used monophasic pills. Participants in the OCP3 group used either monophasic (*n* = 7) or triphasic (*n* = 8) pills. Additional details concerning the amount and type of ethinyl estradiol and progestin taken by OCP users can be found in Williams et al. (2023).

**TABLE 1:** Participant characteristics.

	NAT (*n* = 17)	OCP2 (*n* = 17)	OCP3 (*n* = 15)	*p* value (group)
Age, y	21 ± 2	21 ± 2	21 ± 3	0.848
Weight, kg	66.5 ± 12.7	60.2 ± 8.7	62.6 ± 7.6	0.194
Height, m	1.63 ± 0.09	1.64 ± 0.08	1.64 ± 0.06	0.765
BMI, kg/m^2^ ^a^	25.2 ± 4.6	22.4 ± 3.5	23.2 ± 2.3	0.086
<18.5 (*n*, %)	0, 0%	1, 6%	0, 0%	
18.5–24.9 (*n*, %)	9, 53%	12, 70%	11, 73%	
25.0–29.9 (*n*, %)	6, 35%	4, 24%	4, 27%	
30.0–34.9 (*n*, %)	2, 12%	0, 0%	0, 0%	
≥35.0 (*n*, %)	0, 0%	0, 0%	0, 0%	
Waist circumference, cm	80.3 ± 10.9	75.9 ± 8.8	75.9 ± 6.2	0.265
Hip circumference, cm	104.1 ± 11.2	96.6 ± 7.2	99.5 ± 7.0	0.051
Waist: hip ratio	0.77 ± 0.05	0.78 ± 0.05	0.76 ± 0.04	0.410
VO_2_peak, mL/kg/min	38.2 ± 8.9	40.1 ± 6.9	38.5 ± 7.0	0.740
SBP, mmHg	103 ± 8	106 ± 10	107 ± 5	0.283
DBP, mmHg	62 ± 6	65 ± 8	63 ± 6	0.313
Body composition				
Whole‐body lean mass, kg	41.2 ± 4.7	38.8 ± 4.1	39.5 ± 3.7	0.248
% lean mass	63.1 ± 8.6	66.2 ± 5.3	64.2 ± 7.0	0.436
Whole‐body fat mass, kg	23.1 ± 11.5	18.0 ± 6.0	20.2 ± 6.5	0.216
% body fat	33.2 ± 9.1	29.9 ± 5.7	32.1 ± 7.3	0.425
Bone mineral content, kg	2.4 ± 0.3	2.3 ± 0.3	2.3 ± 0.3	0.416
Menstrual cycle and oral contraceptive pill information				
LH visit date^b^, days	5 ± 1	5 ± 1	5 ± 1	
HH visit date^c^, days	23 ± 3^d^	26 ± 2	26 ± 1	
Menstrual cycle length, days	29 ± 2	—	—	
Ovulation date, days	16 ± 3	—	—	
Duration of OCP use, months	—	40 ± 28	29 ± 23	

*Note*: Data are mean ± SD, unless otherwise indicated.

Abbreviations: BMI, body mass index; DBP, diastolic blood pressure; HH, high hormone; LH, low hormone; NAT, naturally cycling group; OCP2, second‐generation oral contraceptives group; OCP3, third‐generation oral contraceptives group; SBP, systolic blood pressure.

^a^ Chi‐square test *p* = 0.349.

^b^ Early follicular phase or placebo pill use phase.

^c^ Mid‐luteal phase or active pill use phase.

^d^ 7 ± 1 days post‐ovulation with ovulation confirmed using at‐home testing kits.

On average, participants were 21 ± 2 years old, and age did not differ between groups. Average aerobic fitness was in the fair‐to‐good range according to reference values from the American College of Sports Medicine (Medicine ACoS, 2009), and not different between groups. Similarly, SBP and DBP were normal and not different between groups (Table 1).

The average BMI trended to being 11% and 8% lower in the OCP2 and OCP3 groups, respectively, compared with the NAT group (Table 1). Although this difference did not achieve statistical significance, its effect size was medium to large (*p* = 0.086, *η* ^2^ = 0.10). The average BMI was in the normal range (18.5–24.9 kg/m^2^) for the OCP2 and OCP3 groups and in the overweight range (25.0–29.9 kg/m^2^) for the NAT group. Although the distribution of individuals across BMI categories was not different between groups (*p* = 0.349), the only two participants with BMIs in the obese category were in the NAT group. As such, we conducted a sensitivity analysis wherein we removed these 2 participants and re‐ran all statistical analyses. In doing so, we found the primary results of our study unaltered. Therefore, we elected to retain the two participants in question in our dataset. The results of our sensitivity analysis are available as Appendix S1 for the interested reader.

Average hip circumference trended to being ~7% and ~4% lower in the OCP2 and OCP3 groups, respectively, compared with the NAT group. Although this difference did not achieve statistical significance, its effect size was medium to large (*p* = 0.051, *η* ^2^ = 0.12). No significant between‐group differences were observed for waist circumference or any whole‐body DXA measurements (Table 1).

### 3.2 Glucose and insulin

We observed a main effect of group (*p* = 0.025) on fasting glucose concentrations (Figure 3a). Fasting glucose concentrations were ~10% and ~12.5% higher in the OCP2 (4.4 ± 0.3 mM, *p* = 0.002 vs. NAT) and OCP3 (4.5 ± 0.3 mM, *p* < 0.001 vs. NAT) groups, respectively, compared with the NAT group (4.0 ± 0.5 mM). We did not observe any significant group differences in fasting insulin concentrations (Figure 3b) or HOMA‐IR (Figure 3c), and no phase‐based differences in glucose, insulin, or HOMA‐IR.

**FIGURE 3:**
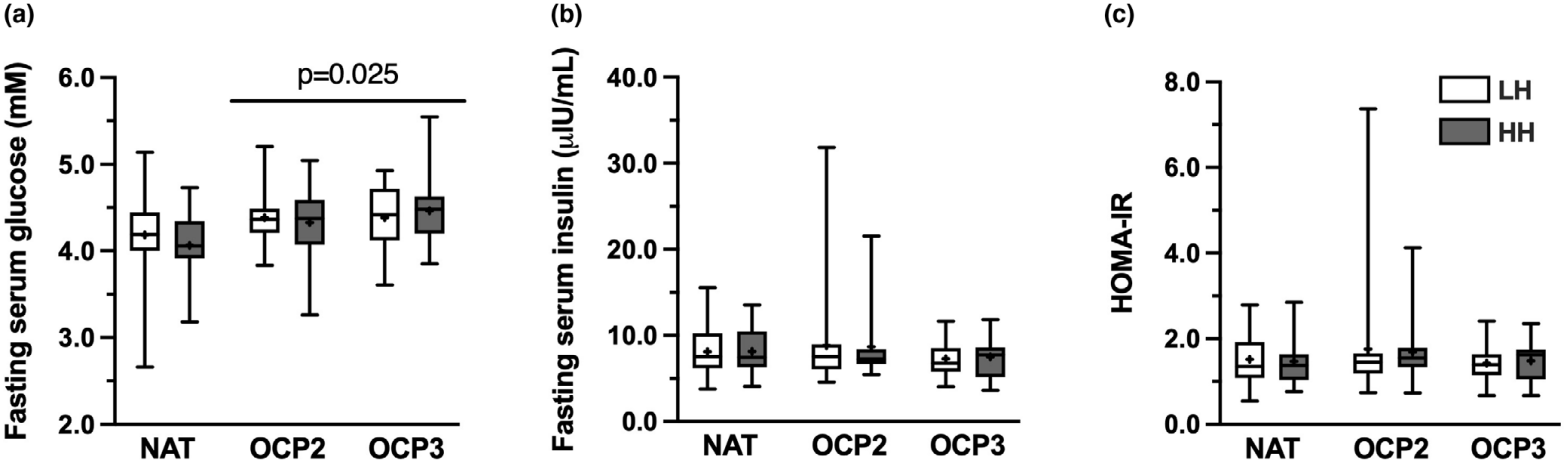
Fasting serum glucose (a) and insulin (b) concentrations, and HOMA‐IR (c). Open (white) boxes represent the LH phase; gray boxes represent the HH phase. Whiskers indicate the minimum and maximum values; boxes and horizontal lines represent the interquartile ranges and medians, respectively. Crosses indicate mean values. HH, high hormone; LH, low hormone; NAT, naturally cycling group; OCP2, second‐generation oral contraceptives group; OCP3, third‐generation oral contraceptives group.

### 3.3 Fasting blood lipids

We observed a main effect of group on fasting TAG (*p* = 0.008) and HDL‐c (*p* = 0.031) concentrations (Table 2). Fasting TAG concentrations were ~56% and ~70% higher in the OCP2 (0.9 ± 0.5 mM, *p* = 0.036 vs. NAT) and OCP3 (0.9 ± 0.3 mM, *p* = 0.011 vs. NAT) groups, respectively, compared with the NAT group (0.6 ± 0.2 mM). Fasting HDL‐c concentrations were ~40% higher in the OCP3 group compared with the OCP2 group (1.8 ± 0.5 mM vs. 1.3 ± 0.5 mM, *p* = 0.010), and there was a trend for higher HDL‐c concentrations in the OCP3 group compared with the NAT group (1.4 ± 0.6 mM, *p* = 0.071 vs. OCP3). The TC/HDL‐c ratio trended to be lower in the OCP3 group compared with the OCP2 group (2.9 ± 1.0 vs. 4.3 ± 2.3, *p* = 0.065). Although this difference did not achieve statistical significance, its effect size was medium to large (*η* ^2^ = 0.12). There were no between‐group differences in TC or LDL‐c, and no phase‐based differences in any fasting blood lipid outcomes.

**TABLE 2:** Fasting blood lipids.

	NAT (*n* = 17)		OCP2 (*n* = 17)		OCP3 (*n* = 15)		*p* value (group)	*p* value (phase)	*p* value (interaction)
	LH	HH	LH	HH	LH	HH			
TAG, mM^a^	0.5 ± 0.2	0.6 ± 0.3	0.9 ± 0.7	0.8 ± 0.4	0.8 ± 0.3	1.0 ± 0.3	**0.008**	0.117	0.103
TC, mM	4.3 ± 0.6	4.4 ± 0.7	4.7 ± 0.9	4.6 ± 0.7	4.8 ± 0.5	4.7 ± 0.6	0.250	0.621	0.137
HDL‐c, mM^a^	1.4 ± 0.6	1.5 ± 0.6	1.3 ± 0.5	1.3 ± 0.5	1.8 ± 0.5	1.7 ± 0.4	**0.031**	0.266	0.104
TC/HDL‐c	3.9 ± 2.1	3.5 ± 1.4	4.2 ± 1.7	4.5 ± 2.9	2.9 ± 1.2	2.8 ± 0.8	0.065	0.575	0.216
LDL‐c, mM	2.7 ± 0.8	2.7 ± 0.8	3.0 ± 0.8	2.9 ± 0.8	2.6 ± 0.6	2.4 ± 0.5	0.187	0.168	0.597

*Note*: Data are mean ± SD. Bolded numbers indicate significant *p*‐values.

Abbreviations: HDL‐c, high‐density lipoprotein cholesterol; HH, high hormone; LDL‐c, low‐density lipoprotein cholesterol; LH, low hormone; mM, mmol/L; NAT, naturally cycling group; OCP2, second‐generation oral contraceptives group; OCP3, third‐generation oral contraceptives group; TAG, triglycerides; TC, total cholesterol.

^a^ Main effect of group (*p* < 0.05).

### 3.4 Regional body composition

ALMI was ~10% lower in both OCP2 and OCP3 groups compared with the NAT group (Table 3). However, when ALM was expressed as a percentage of whole body (*p* = 0.457) or appendicular mass (*p* = 0.388), we found no significant differences between groups. Leg lean mass tended to be 1.3 kg lower in the OCP2 and OCP3 groups compared with the NAT group. Although the difference in leg lean mass did not achieve statistical significance, its associated effect size was medium to large (*p* = 0.055, *η* ^2^ = 0.12). We did not observe any significant between‐group or phase‐based differences in regional fat mass (Table 3).

**TABLE 3:** DXA regional body composition.

	NAT (*n* = 17)	OCP2 (*n* = 17)	OCP3 (*n* = 15)	*p* value (group)
Lean mass				
Trunk, kg	19.3 ± 2.3	18.5 ± 2.0	19.0 ± 1.9	0.595
ALM, kg	19.0 ± 2.6	17.5 ± 2.2	17.4 ± 1.9	0.084
% appendicular mass	60.5 ± 8.1	63.3 ± 4.2	60.7 ± 6.9	0.388
% whole body mass	29.0 ± 3.7	29.8 ± 2.2	28.3 ± 3.4	0.457
ALMI, kg/m^2^	7.2 ± 1.1	6.5 ± 0.8*	6.5 ± 0.5*	**0.016**
Arms, kg	4.1 ± 0.6	3.9 ± 0.5	3.8 ± 0.7	0.481
Legs, kg	14.9 ± 2.1	13.6 ± 1.7	13.6 ± 1.4	0.055
Fat mass				
Trunk, kg	10.3 ± 6.0	8.0 ± 3.7	8.9 ± 3.4	0.346
Android, kg	1.5 ± 1.1	1.1 ± 0.7	1.2 ± 0.6	0.304
Gynoid, kg	4.7 ± 2.3	3.5 ± 1.0	4.1 ± 1.3	0.115
A/G ratio	0.3 ± 0.1	0.3 ± 0.1	0.3 ± 0.1	0.879
AFM, kg	12.0 ± 5.5	9.2 ± 2.4	10.4 ± 3.3	0.141
Arms, kg	2.2 ± 1.0	1.9 ± 0.6	2.1 ± 0.8	0.545
Legs, kg	9.7 ± 4.7	7.3 ± 1.9	8.3 ± 2.6	0.100

*Note*: Data are mean ± SD. Bolded numbers indicate significant *p*‐values.

Abbreviations: AFM, appendicular fat mass; A/G ratio, android/gynoid ratio; ALM, appendicular lean mass; ALMI, appendicular lean mass index; NAT, naturally cycling group; OCP2, second‐generation oral contraceptives group; OCP3, third‐generation oral contraceptives group.

* *p* < 0.05 versus NAT group.

### 3.5 Exploratory sub‐analysis

When participants were pooled into a single group, we observed a moderate negative correlation between ALMI and fasting serum glucose concentrations in the HH phase (*r* = −0.405, *p* = 0.004, Figure 4). However, there was no association between ALMI and fasting serum glucose concentrations in the LH phase (*r* = 0.216, *p* = 0.137).

**FIGURE 4:**
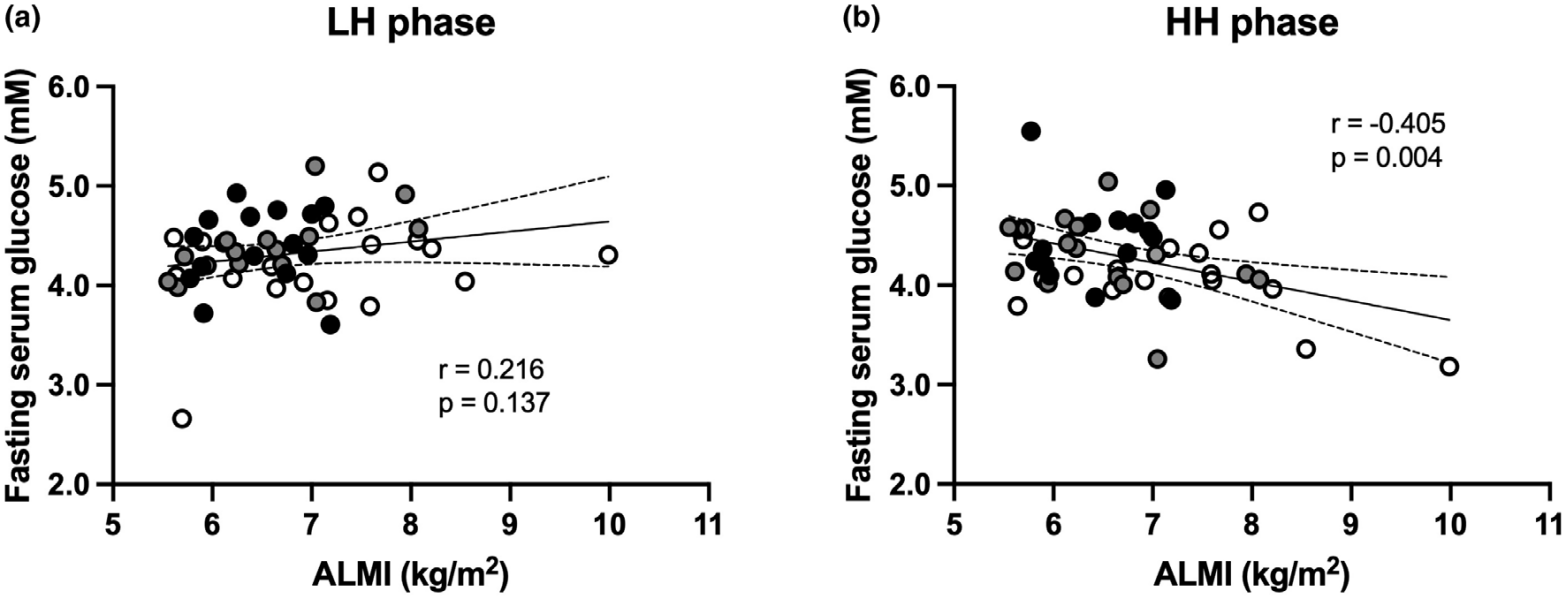
Pearson correlations between ALMI and fasting serum glucose concentrations in the LH (a) and HH (b) phases. Statistics were run on pooled data. However, for clarity, white (open) circles represent the NAT group, gray circles represent the OCP2 group, and black circles represent the OCP3 group. The solid lines are the lines of best fit, and the dashed lines are the associated 95% confidence intervals. ALMI, appendicular lean mass index; HH, high hormone; LH, low hormone; NAT, naturally cycling group; OCP2, second‐generation oral contraceptives group; OCP3, third‐generation oral contraceptives group.

## 4 DISCUSSION

The main finding of this study was that fasting serum glucose and triglyceride concentrations were ~10% and ~55%–70% higher, respectively, in OCP users of both generations compared with naturally cycling young females, which aligned with our hypothesis. We also found that appendicular lean mass, when normalized to height squared, was ~10% lower in OCP users of both generations compared with naturally cycling participants. But, this effect disappeared when ALM was expressed as a percentage of body mass. Third‐generation OCP users also had higher HDL‐c concentrations compared to second‐generation OCP users. In agreement with our hypothesis, we found that hormone phase (low vs. high) did not appear to affect fasting features of glucose and lipid metabolism or body composition. However, our exploratory sub‐analysis revealed an inverse correlation between ALMI and fasting glucose concentrations in the high—but not low—hormone phase when all participants were pooled into a single group. This suggests that hormone phase may exert a moderate level of influence over glucose homeostasis, but more adequately powered work is needed. Considering that over half of Canadian women will use OCPs at some point in their lives (Black et al., 2015; Rotermann et al., 2015), our findings underscore the need to better understand the subtle, yet significant, impacts that OCPs exert on metabolic health.

### 4.1 Altered fasting glucose and lipid profiles in OCP users

This is the first study to report that fasting glucose and triglyceride concentrations were higher among users of the most commonly prescribed OCP formulations in Canada (Black et al., 2015; Rotermann et al., 2015) compared with NAT and that fasting HDL‐c concentrations were higher in third versus second‐generation OCP users. Our findings align with two previous studies (Frempong et al., 2008; Morin‐Papunen et al., 2008) that observed modestly greater triglyceride and HDL‐c concentrations in OCP users compared to control groups. The differences in TAG concentrations between the NAT and OCP groups may be underpinned by differences in hormone profile (which we expand upon in the next section), diet, or ethnicity. Dietary fats have distinct effects on circulating lipids: for example, omega‐3 fatty acid and saturated fat intake are associated with lower and higher TAG concentrations, respectively. Similarly, certain ethnic groups may present with lower (Chinese) or higher (Asian Indians) circulating TAG concentrations (Frank et al., 2014). However, we did not control for or assess diet or ethnicity, which was a limitation of this study. While neither of these prior studies (Frempong et al., 2008; Morin‐Papunen et al., 2008) observed differences in fasting glucose concentrations, Morin‐Papunen et al. (2008) found that fasting insulin and indices of insulin resistance (HOMA%S and HOMA%B) were higher among OCP users compared to a reference group of females who did not report using any form of hormonal contraception. Likewise, Frempong et al. (2008) found that fasting insulin trended (*p* = 0.05) to being higher in OCP users compared to females who did not use OCPs, which suggests that OCP use may have a negative, albeit slight, impact on glucose metabolism. In contrast, other studies have reported no difference (Isacco et al., 2014; Nessa et al., 2005; Straznicky et al., 1998), or even improvements (Kim et al., 2002; Troisi et al., 2000), in fasting glucose and lipids in OCP users compared to control groups. The precise reason behind this lack of consensus in the literature is unknown, but a possible contributing factor is the heterogeneity of comparator groups. Many previous studies did not report rigorously controlling for cycle length, regularity, or prior exposure to hormonal contraceptives, which may affect our interpretation of their results. Further, it is possible that lifestyle factors, including physical activity, diet, sleep, or stress could differ between groups and would warrant future investigation to incorporate into examinations of glucose metabolism between OCP and NAT groups.

Another potential factor contributing to discrepancies in the literature is the range of OCPs used by participants across studies. For example, in the two studies (Kim et al., 2002; Troisi et al., 2000) that demonstrated a favorable association between OCP use and fasting features of glucose metabolism, the majority (88%) of participants were on first‐generation OCPs that contained a comparatively high dose of progestin (1 mg norethindrone). On the other hand, all participants in the studies that did not detect between‐group differences (Isacco et al., 2014; Nessa et al., 2005; Straznicky et al., 1998) used second‐ or third‐generation OCPs. These studies, however, either did not report the pill cycle day(s) on which blood samples were obtained (Nessa et al., 2005; Straznicky et al., 1998) or employed a very wide date range (i.e., blood samples were obtained any time during the 21 days of active pill use) (Isacco et al., 2014). Recent work suggests that, contrary to previous assumptions, circulating concentrations of synthetic and endogenous estradiol vary substantially over the 3 weeks of active pill use in a monophasic OCP cycle (Rodriguez et al., 2024). Given that estradiol is known to directly promote insulin sensitivity and lipid oxidation (Alemany, 2021), it may be important to control for the bioavailability of this hormone when measuring outcomes related to glucose and lipid metabolism. Specifically, drawing blood samples over a narrower date range (e.g., days 2–7 of the third week of active pill use, as we did in the present study) may help ensure that circulating concentrations of estradiol are consistent across participants and increase the precision of detecting between‐group differences in metabolic outcomes. In the present study, we implemented rigorous temporal controls over blood sampling resulting in tightly controlled endogenous estradiol and progesterone levels in OCP groups, as previously reported in Williams et al. (2024).

Our results add to the body of literature describing the detrimental effects of OCPs on fasting features of glucose and lipid metabolism. Elevations in blood glucose and triglycerides (and decrements in HDL‐c) are generally undesirable and, over time, may increase an individual's risk for developing cardiometabolic conditions, such as MetS (Alberti et al., 2009). On average participants in this study did not have MetS: we observed healthy average fasting blood glucose (<5.6 mM), waist circumference (<88 cm), blood pressure (SBP <130 mmHg and/or DBP < 85 mmHg), and lipid partitioning (fasting triglycerides <1.7 mM; HDL‐c ≥1.3 mM) across all three groups. Only one individual (in the OCP2 group) met the criteria for MetS. Furthermore, the magnitude of the differences in fasting glucose (0.4–0.5 mM) and triglycerides (0.3 mM) that we observed between the NAT and OCP groups, while statistically significant, were small. This suggests that for young, healthy, normal‐weight females, OCP use may not meaningfully increase the likelihood of developing MetS. It is possible, however, that these modest yet significant differences in blood biochemistry may be clinically relevant for individuals with preexisting risk factors, such as higher BMI. For example, for someone whose fasting blood glucose was 5.1 mM prior to starting an OCP, a 0.5 mM increase would reclassify them from “healthy” (<5.6 mM) to “elevated” (≥5.6 mM). This speculation remains to be confirmed in future work on individuals at increased risk for metabolic disease. Given that the prevalence of MetS is increasing worldwide (Becker et al., 2021), particularly among women 18–45 years (the demographic most likely to use OCPs (Ford et al., 2004)), additional studies considering the intersections of OCP use and MetS are warranted.

### 4.2 Potential mechanisms underpinning OCP‐related differences in metabolic profile

In the present study, we observed that OCP use (regardless of generation) was associated with higher fasting glucose and triglyceride concentrations. Others (Grandi et al., 2016; Stanczyk et al., 2013) have postulated that the synthetic estrogen (ethinyl estradiol) contained in second‐ and third‐generation OCPs may be associated with these undesirable metabolic effects. Indeed, the few OCPs that provide *endogenous* estrogen (17β estradiol)—though far less commonly prescribed due to the increased incidence of breakthrough bleeding—appear less likely to negatively impact glucose and lipid metabolism (Grandi et al., 2014; Junge et al., 2012). Indeed, a robust body of rodent and cell work shows that 17β estradiol is intricately and beneficially involved in various relevant aspects of energy metabolism (Alemany, 2021). Among other actions, this hormone (1) facilitates insulin secretion (Bian et al., 2019), (2) stimulates glucose uptake from the bloodstream (Garrido et al., 2013), (3) reduces hepatic glucose release (Devries et al., 2005), (4) promotes a shift from lipid deposition (as adipose tissue) to oxidation (Litwak et al., 2014), and (5) reduces circulating concentrations of triglycerides (Luo et al., 2017). Endogenous estrogen production is dramatically suppressed by chronic exposure to ethinyl estradiol, and we have previously published data to confirm that circulating concentrations of 17β estradiol were significantly lower in both OCP groups (OCP2: 131 ± 153 pmol/L, OCP3: 75 ± 15 pmol/L) versus NAT group (531 ± 186 pmol/L, *p* < 0.010) during the HH phase in the present study (Williams et al., 2024). It is, therefore, possible that OCP users had less 17β estradiol available to inhibit hepatic glucose release and promote lipid oxidation, thereby resulting in increased fasting glucose and triglyceride concentrations, respectively. However, this is speculation that remains to be confirmed in human intervention studies.

Our finding that HDL‐c concentrations were higher in OCP3 versus OCP2 users may relate to the type of progestin used in each drug. Ethinyl estradiol alone is associated with increased HDL‐c and triglyceride and decreased LDL‐c concentrations (Morin‐Papunen et al., 2008; Oster et al., 1982). When combined with androgenic progestins (found in second‐generation OCPs), this progestin may antagonize (Endrikat et al., 2002; Oster et al., 1982) the effects of ethinyl estradiol on serum lipids, while less androgenic progestins (found in third‐generation OCPs) may agonize their effects (van Rooijen et al., 2002). Studies in OCP3 users report increased HDL‐c and triglycerides (and decreased LDL‐c, which we did not observe) but, to our knowledge, this is not something reported in OCP2 users (Kemmeren et al., 2001). Consistent with this idea, previous work has shown that non‐ or antiandrogenic fourth generation progestins like dienogest (Wiegratz et al., 2002), drospirenone (Oelkers et al., 1995), and chlormadinone acetate (Cagnacci et al., 2009; Grandi et al., 2014; Grandi et al., 2015) alter lipid partitioning to an even greater extent than the less androgenic progestins contained in OCP3s. The diverse and opposing effects that different classes of progestins have on lipid metabolism highlight the importance of collecting information on, or controlling for, the generation and even the specific formulation of OCP in future work. Further, the apparently divergent interactions between ethinyl estradiol and second‐ and third‐generation progestins may help explain why there remains no consensus on the effect of OCPs on metabolism.

### 4.3 Distinct body composition in OCP users versus NAT

Differences in the hormonal milieu between the NAT, OCP2, and OCP3 groups likely underpin our metabolic findings, at least to some degree. However, an alternative or additional explanation for the distinct metabolic profile that we observed in OCP users compared with naturally cycling females might be attributed to differences in body composition. Compared with the OCP groups, the NAT group had significantly higher ALMI and tended to have higher BMI, hip circumference, and leg lean mass. But this effect disappeared when ALM was expressed as a percentage of body mass. Taken together, these results suggest that individuals in the NAT group may have had larger and leaner lower bodies. As a major glucose reservoir, skeletal muscle plays a key role in glucose homeostasis and is responsible for as much as 75% of glucose uptake from the bloodstream following a carbohydrate‐containing meal (DeFronzo et al., 1981).

In the present study, we also observed that ALMI (a proxy of skeletal muscle mass) was inversely related to fasting glucose concentrations during the HH phase. This means that individuals with higher ALMI were more likely to have lower fasting blood glucose. While fasting glucose concentrations are typically a reflection of hepatic glucose output rather than skeletal muscle glucose uptake (Ter Horst et al., 2016), others have observed an inverse relationship between skeletal muscle mass and fasting blood glucose. For example, in a sample of 587 adults 26–95 years of age, Kalyani et al. (2012) reported that skeletal muscle mass index (muscle cross‐sectional area by computed tomography divided by body weight squared) correlated negatively with fasting glucose concentrations. Buscemi et al. (2021) also reported that sarcopenia and low appendicular lean mass predict impaired fasting glucose in elderly women. These findings suggest that fasting glucose concentrations may not be exclusively determined by hepatic glucose output and that skeletal muscle may contribute in some fashion. It is possible that individuals with greater quantities of skeletal muscle can remove more glucose from the bloodstream in the postprandial and fasted states. However, given the cross‐sectional nature of our study, it is not possible to infer causality between ALMI and fasting glucose in this sample. Similarly, we cannot necessarily conclude that OCP use is related to the between‐group differences in body composition that we observed. The fact that we failed to detect between‐group differences in ALM when expressed as a percentage of whole‐body and appendicular body mass (rather than height) suggests that lean mass may be unrelated to the metabolic differences we observed between NAT females and the OCP groups. Regardless, given the known relationship between body composition and features of glucose and lipid metabolism, future studies must ensure that groups are matched for lean and fat mass to better isolate the potential effects of OCPs on the metabolic outcomes of interest.

### 4.4 Limitations

A limitation of the present study is that we did not control for participants' fitness level. Further, this study did not assess the physical activity habits of participants, including types of habitual physical activity or structured exercise training participants performed. However, since VO_2_peak values were not significantly different between groups, aerobic fitness likely did not contribute to the observed between‐group differences in metabolism and body composition; though it is possible that discrepancies in lean body mass in this study could be attributed to differences in physical activity habits distinct from aerobic fitness, such as resistance training, and warrants further investigation. Furthermore, this study may be vulnerable to sample bias, as – anecdotally – most studies conducted in our Department tend to attract healthy, fit individuals who are interested in learning more about their own health status. Since this study was a secondary analysis from a larger study designed to examine the effect of OCPs on vascular function, we were underpowered to detect differences in most metabolic and body composition outcomes. Our null findings and observed trends should, therefore, be confirmed in future, adequately powered studies. Lastly, recent work has challenged the generally accepted assumption that exogenous and endogenous estradiol availability is relatively stable during monophasic OCP use (Rodriguez et al., 2024). Given the interindividual and within‐pill cycle variability that Rodriguez et al. (2024) report, it may be important to measure and present the ratio of 17β estradiol to ethinyl estradiol in OCP users at key time points. Further, the inclusion of both triphasic and monophasic third‐generation OCP users may have introduced additional variability to this group; however, triphasic users were assessed during their highest hormone week and use of both triphasic and monophasic OCP formulations reflects the continued prescription recommendations in Canada (Black, Guilbert, Costescu, et al., 2015).

## 5 CONCLUSION

We found that OCP users had higher fasting serum glucose and triglyceride concentrations and lower ALMI compared with NAT young females. With the exception of HDL‐c (which was higher in the OCP3 vs. OCP2 group), we found that neither OCP generation nor hormone phase had a notable impact on most fasting features of glucose and lipid metabolism. This suggests that—depending on the outcomes of interest—it may be appropriate for researchers to expand their eligibility criteria for female participants to include both naturally cycling individuals as well as OCP users. Ultimately, these expanded inclusion criteria would help increase female participation in physiological research and thereby increase the relevance and generalizability of the research. Additional work is needed to explore the effects of OCPs on postprandial glucose and lipid metabolism and in individuals at higher risk for metabolic disease (such as those with overweight/obesity or MetS). Lastly, given the sharp increase in the popularity of hormonal intrauterine devices and other long‐acting hormonal contraceptives over the past decade, future work should examine how these low‐dose, local hormonal contraceptives may influence metabolism and body composition.

## AUTHOR CONTRIBUTIONS

**Claire Karaguesian:** Conceptualization; data curation; formal analysis; visualization. **Jennifer S. Williams:** Data curation; formal analysis; project administration. **Maureen J. MacDonald:** Funding acquisition; project administration; resources; supervision. **Kirsten E. Bell:** Conceptualization; formal analysis; funding acquisition; project administration; supervision.

## FUNDING INFORMATION

This work was supported by McMaster's Kinesiology Research Award to CK, a Natural Sciences and Engineering Research Council (NSERC) Discovery Grant to MJM (PG‐PIN‐2019 05413, CGSD3‐534327‐2019), and a NSERC Canadian Graduate Scholarship (Doctoral) and Ontario Women's Health Scholars Awarded funded by the Ontario Ministry of Health and Ministry of Long‐Term Care to JSW.

## CONFLICT OF INTEREST STATEMENT

The authors have nothing to disclose.

## ETHICS STATEMENT

This study conforms to the standards set by the Declaration of Helsinki, apart from registration as a clinical trial, and was approved by the Hamilton Integrated Research Ethics Board (HIREB #7827). Written informed consent was obtained from all volunteers prior to their participation. Separate consent to publish an image was obtained from the individual depicted in Figure 2.

## Supporting information


Appendix S1.

## Data Availability

Some or all datasets generated during and/or analyzed during the current study are not publicly available but are available from the corresponding author on reasonable request.
