# Supplementary material for: Elevated fasting glucose and triglycerides in combined oral contraceptive users compared to naturally cycling females
Source: Physiol Rep. 2026 Oct 12;14(19):e71135. doi: 10.14814/phy2.71135 (PMC13659045; doi:10.14814/phy2.71135)
Supplement: Supplementary file 1 [file PHY2-14-e71135-s001.docx]

**Supplementary Material**

This document provides the results of a sensitivity analysis wherein we removed the two participants in the NAT group with BMIs in the obese category. The primary findings of our study were unaltered by excluding these individuals.

**Table S1.** Participant characteristics.

|  | **NAT**  **(n=15)** | **OCP2**  **(n=17)** | **OCP3**  **(n=15)** | **p-value (group)** |
| --- | --- | --- | --- | --- |
| Age, y | 21 ± 2 | 21 ± 2 | 21 ± 3 | 0.863 |
| Weight, kg | 63.1 ± 8.0 | 60.2 ± 8.7 | 62.6 ± 7.6 | 0.556 |
| Height, m | 1.62 ± 0.09 | 1.64 ± 0.08 | 1.64 ± 0.06 | 0.682 |
| BMI, kg/m^2 d^ | 24.0 ± 3.6 | 22.4 ± 3.5 | 23.2 ± 2.3 | 0.351 |
| < 18.5 (n, %) | 0, 0% | 1, 6% | 0, 0% |  |
| 18.5–24.9 (n, %) | 9, 60% | 12, 70% | 11, 73% |  |
| 25.0–29.9 (n, %) | 6, 40% | 4, 24% | 4, 27% |  |
| 30.0–34.9 (n, %) | 0, 0% | 0, 0% | 0, 0% |  |
| ≥ 35.0 (n, %) | 0, 0% | 0, 0% | 0, 0% |  |
| Waist circumference, cm | 78.3 ± 9.7 | 75.9 ± 8.8 | 75.9 ± 6.2 | 0.662 |
| Hip circumference, cm | 101.8 ± 9.6 | 96.6 ± 7.2 | 99.5 ± 7.0 | 0.190 |
| Waist:hip ratio | 0.77 ± 0.05 | 0.78 ± 0.05 | 0.76 ± 0.04 | 0.407 |
| VO_2_peak, mL/kg/min | 39.6 ± 8.4 | 40.1 ± 6.9 | 38.5 ± 7.0 | 0.835 |
| SBP, mmHg | 102 ± 8 | 106 ± 10 | 107 ± 5 | 0.228 |
| DBP, mmHg | 61 ± 5 | 65 ± 8 | 63 ± 6 | 0.173 |
| *Body composition* | | | | |
| Whole-body lean mass, kg | 40.8 ± 4.6 | 38.8 ± 4.1 | 39.5 ± 3.7 | 0.391 |
| % lean mass | 64.9 ± 7.3 | 66.2 ± 5.3 | 64.2 ± 7.0 | 0.684 |
| Whole-body fat mass, kg | 20.5 ± 9.1 | 18.0 ± 6.0 | 20.2 ± 6.5 | 0.563 |
| % body fat | 31.3 ± 7.7 | 29.9 ± 5.7 | 32.1 ± 7.3 | 0.656 |
| Bone mineral content, kg | 2.4 ± 0.3 | 2.3 ± 0.3 | 2.3 ± 0.3 | 0.456 |
| *Menstrual cycle and oral contraceptive pill information* | | | | |
| LH visit date^a^, days | 5 ± 1 | 5 ± 1 | 5± 1 |  |
| HH visit date^b^, days | 23 ± 3^c^ | 26 ± 2 | 26 ± 1 |  |
| Menstrual cycle length, days | 29 ± 2 | – | – |  |
| Ovulation date, days | 16 ± 3 | – | – |  |
| Duration of OCP use, months | – | 40 ± 28 | 29 ± 23 |  |

Data are mean ± SD, unless otherwise indicated. Abbreviations: BMI, body mass index; DBP, diastolic blood pressure; HH, high hormone; LH, low hormone; NAT, naturally cycling group; OCP2, second-generation oral contraceptives group; SBP, systolic blood pressure; OCP3, third-generation oral contraceptives group.
^a^Early follicular phase or placebo pill use phase
^b^Mid-luteal phase or active pill use phase
^c^7 ± 1 days post-ovulation with ovulation confirmed using at-home testing kits
^d^Chi-square test p = 0.349

**Table S2.** Fasting blood lipids.

|  | **NAT**  **(n=15)** | | **OCP2**  **(n=17)** | | **OCP3**  **(n=15)** | | **p-value**  **(group)** | **p-value**  **(phase)** | **p-value**  **(interaction)** |
| --- | --- | --- | --- | --- | --- | --- | --- | --- | --- |
|  | **LH** | **HH** | **LH** | **HH** | **LH** | **HH** |  |  |  |
| TAG, mM^a^ | 0.5 ± 0.2 | 0.6 ± 0.3 | 0.9 ± 0.7 | 0.8 ± 0.4 | 0.8 ± 0.3 | 1.0 ± 0.3 | **0.018** | 0.132 | 0.114 |
| TC, mM | 4.2 ± 0.6 | 4.4 ± 0.7 | 4.7 ± 0.9 | 4.6 ± 0.7 | 4.8 ± 0.5 | 4.7 ± 0.6 | 0.182 | 0.684 | 0.137 |
| HDL-c, mM^a^ | 1.4 ± 0.6 | 1.6 ± 0.6 | 1.3 ± 0.5 | 1.3 ± 0.5 | 1.8 ± 0.5 | 1.7 ± 0.4 | **0.034** | 0.268 | 0.120 |
| TC/HDL-c | 3.6 ± 2.0 | 3.2 ± 1.3 | 4.2 ± 1.7 | 4.5 ± 2.9 | 2.9 ± 1.2 | 2.8 ± 0.8 | 0.057 | 0.671 | 0.295 |
| LDL-c, mM | 2.5 ± 0.7 | 2.5 ± 0.8 | 3.0 ± 0.8 | 2.9 ± 0.8 | 2.6 ± 0.6 | 2.4 ± 0.5 | 0.116 | 0.199 | 0.579 |

Data are mean ± SD. Abbreviations: LH, low hormone; HH, high hormone; HDL-c, high-density lipoprotein cholesterol; LDL-c, low-density lipoprotein cholesterol; TC, total cholesterol; TAG, triglycerides; NAT, naturally cycling group; OCP2, second-generation oral contraceptives group; OCP3, third-generation oral contraceptives group; mM, mmol/L.
^a^Main effect of group (p < 0.05)

**Table S3**. DXA regional body composition.

|  | **NAT**  **(n=15)** | **OCP2**  **(n=17)** | **OCP3**  **(n=15)** | **p-value**  **(group)** |
| --- | --- | --- | --- | --- |
| *Lean mass* | | | | |
| Trunk, kg | 19.2 ± 2.4 | 18.5 ± 2.0 | 19.0 ± 1.9 | 0.655 |
| ALM, kg | 18.7 ± 2.4 | 17.5 ± 2.2 | 17.4 ± 1.9 | 0.214 |
| % appendicular mass | 62.1 ± 7.1 | 63.3 ± 4.2 | 60.7 ± 6.9 | 0.495 |
| % whole body mass | 29.6 ± 3.4 | 29.8 ± 2.2 | 28.3 ± 3.4 | 0.362 |
| ALMI, kg/m^2^ | 7.2 ± 1.2 | 6.5 ± 0.8* | 6.5 ± 0.5* | **0.047** |
| Arms, kg | 4.0 ± 0.7 | 3.9 ± 0.5 | 3.8 ± 0.7 | 0.626 |
| Legs, kg | 14.6 ± 1.9 | 13.6 ± 1.7 | 13.6 ± 1.4 | 0.161 |
| *Fat mass* | | | | |
| Trunk, kg | 9.0 ± 4.9 | 8.0 ± 3.7 | 8.9 ± 3.4 | 0.754 |
| Android, kg | 1.3 ± 0.9 | 1.1 ± 0.7 | 1.2 ± 0.6 | 0.674 |
| Gynoid, kg | 4.2 ± 1.8 | 3.5 ± 1.0 | 4.1 ± 1.3 | 0.210 |
| A/G ratio | 0.3 ± 0.1 | 0.3 ± 0.1 | 0.3 ± 0.1 | 0.983 |
| AFM, kg | 10.7 ± 4.4 | 9.2 ± 2.4 | 10.4 ± 3.3 | 0.417 |
| Arms, kg | 2.0 ± 0.7 | 1.9 ± 0.6 | 2.1 ± 0.8 | 0.749 |
| Legs, kg | 8.7 ± 3.7 | 7.3 ± 1.9 | 8.3 ± 2.6 | 0.332 |

Data are mean ± SD. Abbreviations: AFM, appendicular fat mass; A/G ratio, android/gynoid ratio; ALM, appendicular lean mass; ALMI, appendicular lean mass index; NAT, naturally cycling group; OCP2, second-generation oral contraceptives group; OCP3, third-generation oral contraceptives group.
*p < 0.05 vs. NAT group

**Figure S1. Fasting serum glucose (A) and insulin (B) concentrations, and HOMA-IR (C).** Open (white) boxes represent the LH phase; grey boxes represent the HH phase. Whiskers indicate the minimum and maximum values; boxes and horizontal lines represent the interquartile ranges and medians, respectively. Crosses indicate mean values. Abbreviations: LH, low hormone; HH, high hormone; NAT, naturally cycling group; OCP2, second-generation oral contraceptives group; OCP3, third-generation oral contraceptives group.

**Figure S2. Pearson correlations between ALMI and fasting serum glucose concentrations in the LH (A) and HH (B) phases.** Statistics were run on pooled data. However, for clarity, white (open) circles represent the NAT group, grey circles represent the OCP2 group, and black circles represent the OCP3 group. The solid lines are the lines of best fit, and the dashed lines are the associated 95% confidence intervals. Abbreviations: ALMI, appendicular lean mass index; HH, high hormone; LH, low hormone; NAT, naturally cycling group; OCP2, second-generation oral contraceptives group; OCP3, third-generation oral contraceptives group.
